# Small cell carcinoma of the urinary bladder and prostate: Cytological analyses of four cases with emphasis on the usefulness of cytological examination

**DOI:** 10.3892/ol.2013.1715

**Published:** 2013-11-29

**Authors:** KEIKO YOSHIDA, MITSUAKI ISHIDA, AKIKO KAGOTANI, NOZOMI IWAMOTO, MUNEO IWAI, HIDETOSHI OKABE

**Affiliations:** Division of Diagnostic Pathology and Department of Clinical Laboratory Medicine, Shiga University of Medical Science, Otsu, Shiga 520-2192, Japan

**Keywords:** small cell carcinoma, urinary bladder, prostate, urine cytology

## Abstract

The occurrence of small cell carcinoma in the urinary bladder and prostate is rare. Only a few reports on the cytological features of small cell carcinoma of the urinary bladder in the urine specimen have been documented and, moreover, the urinary cytological features of prostate small cell carcinoma have been rarely reported. In this study, we analyzed the cytological features of four cases of small cell carcinoma of the urinary bladder and prostate, and discussed the usefulness of cytological examination of urine specimen for this type of tumor. This study included two urinary bladder and two prostate small cell carcinoma cases. Analyses of the cytological features of these cases revealed the following: i) the background was mostly inflammatory and necrotic material was also occasionally observed; ii) numerous tumor cells were present in two cases, whereas only a few neoplastic cells were observed in the remaining cases; iii) the neoplastic cells were small in size, had scant cytoplasm and a high nuclear/cytoplasmic ratio, and were arranged in small clusters or occasionally as single cells; iv) the tumor cell clusters showed prominent nuclear moldings; and v) the nuclei of the neoplastic cells were round to oval in shape with finely granular chromatin containing inconspicuous nucleoli. The cytological features of small cell carcinoma in the urine specimen are characteristic. Therefore, careful observation of the urine specimen may lead to a correct diagnosis of small cell carcinoma of the urinary bladder and, moreover, cytodiagnosis of prostate small cell carcinoma may also be possible.

## Introduction

Small cell carcinoma is one of the histopathological subtypes that demonstrates aggressive clinical behavior and most commonly arises in the lung. However, albeit rare, it can occur at extrapulmonary sites, such as the gastrointestinal tract, pancreatobiliary system, salivary gland, uterine cervix and urinary tract. Extrapulmonary small cell carcinoma comprises ~4% of all small cell carcinomas. Extrapulmonary small cell carcinomas share histopathological and immunohistochemical features with their pulmonary counterpart. The tumor cells are small in size and relatively uniform. They have scant cytoplasm, powdery chromatin and inconspicuous nucleoli. Mitotic figures and apoptotic bodies are commonly observed, and the presence of geographic necrosis is also characteristic. Immunohistochemically, the neoplastic cells show positive immunoreactivity for neuroendocrine markers, such as chromogranin A, synaptophysin and CD56 (1,2).

The occurrence of small cell carcinoma is rare in the urinary bladder and prostate, and its incidence is reported to be 0.3–0.7% and 0.5–2% of all primary carcinomas of the urinary bladder and prostate, respectively (1,2). Although the histopathological features of small cell carcinoma of the urinary bladder are well-recognized, only a few reports on the cytological features of the urine specimen of this type of tumor have been documented (3–8). Moreover, the urinary cytological features of small cell carcinoma of the prostate have rarely been reported (9). In this retrospective study, we analyzed the urinary cytological features of small cell carcinoma of the urinary bladder and prostate and discuss the usefulness of cytological examination of urine specimen for this type of tumor.

## Materials and methods

Urine specimens from patients at Shiga University of Medical Science Hospital (Shiga, Japan) diagnosed histopathologically as small cell carcinoma of the urinary bladder or prostate were retrieved. Four urine specimens from four patients were available in this study. The specimens were all voided urine samples that were obtained preceding the patients' surgical procedure or cystoscopy. The cytological specimens were Papanicolaou-stained and analyzed for cytological features, including background, number of neoplastic cells, cellular arrangement, cell size and shape, as well as nuclear features.

Tissues from cystoscopic, surgical resections or biopsy were fixed by formalin and embedded in paraffin. Tissue sections were stained with hematoxylin and eosin, and subjected to immunohistochemistry using an autostainer (BenchMark XT system; Ventana Medical Systems Inc., Tucson, AZ, USA) according to the manufacturer's instructions. The following primary antibodies were used: Mouse monoclonal antibody against CD56 (CD564; Novocastra Laboratories, Ltd., Newcastle upon Tyne, UK), mouse monoclonal antibody against chromogranin A (DAK-A3; DAKO Cytomation, Glostrup, Denmark) and mouse monoclonal antibody against synaptophysin (27G12; Novocastra Laboratories, Ltd.). The study was approved by the ethics committee of Shiga University of Medical Science (Shiga, Japan). Written informed consent was obtained from the patients.

## Results

### Clinicopathological features

Table I summarizes the clinicopathological features of four cases of small cell carcinoma of the urinary bladder and prostate. This study included two urinary bladder and two prostate cases. All cases were elderly men (average age, 74 years; range, 64–82 years). Multiple liver metastases (case 1) and direct urinary bladder invasion (cases 3 and 4) were detected by imaging analyses.

### Cytological findings

**Figure 1:**
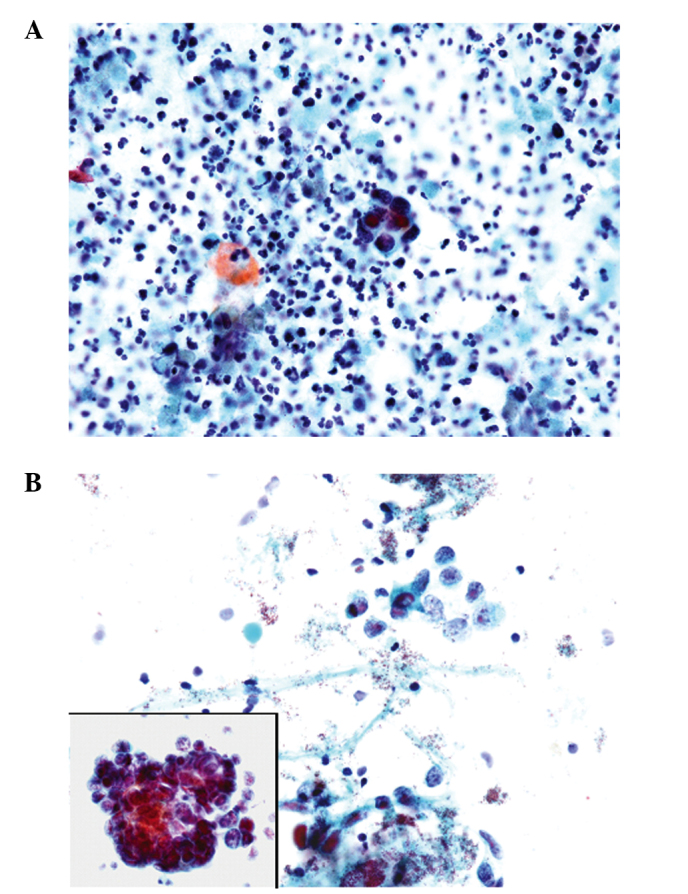
Cytological findings of small cell carcinoma of the urinary bladder and prostate. (A) Small cell carcinoma of the urinary bladder (case 2). Small clusters of tumor cells with scant cytoplasm and a high nuclear/cytoplasmic ratio, and with granular chromatin without nucleoli, in an inflammatory and necrotic background (Papanicolaou stain; magnification, ×400). (B) Small cell carcinoma of the prostate (case 4). Single tumor cells and clusters of tumor cells showing nuclear molding (inset) are observed. These neoplastic cells have scant cytoplasm and granular chromatin without nucleoli (Papanicolaou stain; magnification, ×400; inset, ×400).

The cytological features of the four cases are summarized in Table I. The background of the urine specimens was mostly inflammatory, in which numerous neutrophils were present. Necrotic material was also occasionally observed in cases 2–4 (Fig. 1A), whereas the background was clean in case 1. A number of tumor cells were observed in one urinary bladder case (case 1) and one prostate case (case 3); however, only a few neoplastic cells were present in cases 2 and 4. The neoplastic cells were small in size, had scant cytoplasm and a high nuclear/cytoplasmic ratio, and were arranged in small clusters or occasionally as single cells (Fig. 1A and B). The tumor cell clusters demonstrated prominent nuclear molding (Fig. 1B). The nuclei of the neoplastic cells were round to oval in shape with finely granular chromatin containing inconspicuous nucleoli (Fig. 1A and B). Neither conventional urothelial carcinoma nor adenocarcinoma components were observed in the cytological specimens of any of the cases.

### Histopathological findings

**Figure 2:**
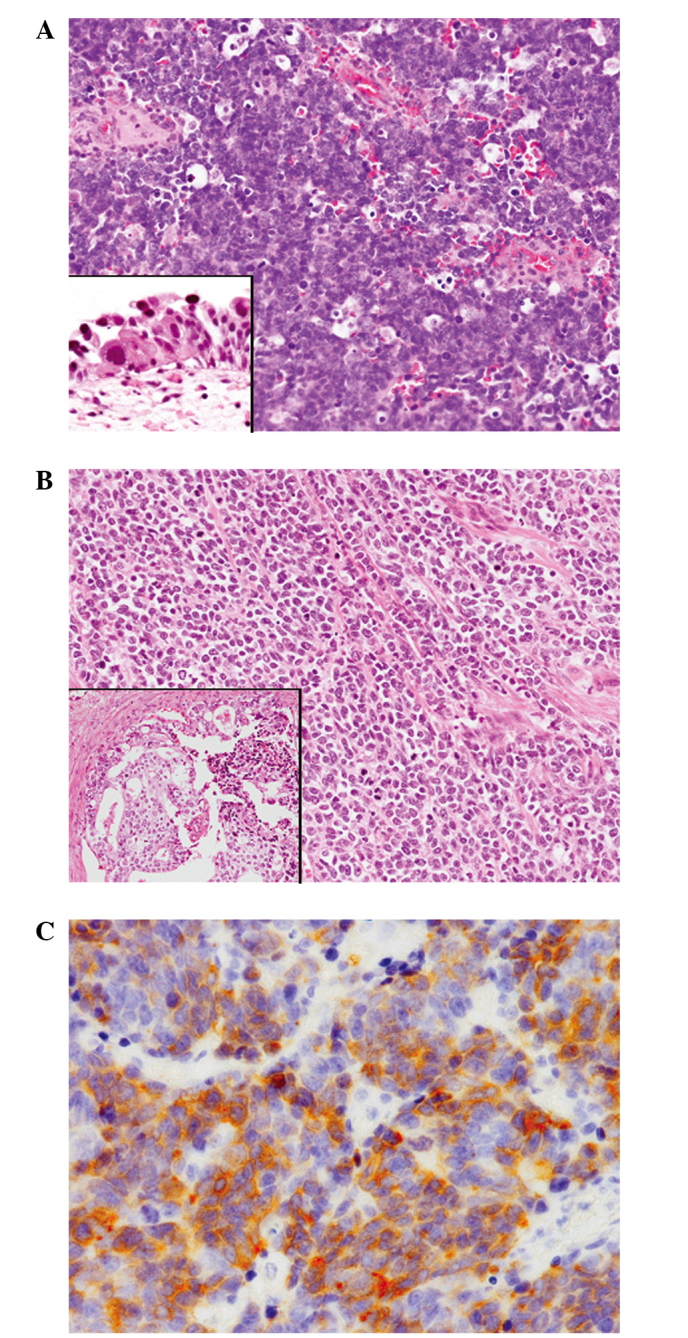
Histopathological and immunohistochemical findings of small cell carcinoma of the urinary bladder and prostate. (A) Small cell carcinoma of the urinary bladder (case 1). Diffuse proliferation of small-sized cells with scant cytoplasm and round to oval hyperchromatic nuclei. Apoptotic bodies and mitotic figures were frequently observed. A carcinoma *in situ* component was also present (inset) (hematoxylin and eosin; magnification, ×200; inset, ×400). (B) Small cell carcinoma of the prostate (case 3). Diffuse proliferation of small-sized cells. A conventional adenocarcinoma component was also observed (inset) (hematoxylin and eosin; magnification, ×100; inset, ×100). (C) Synaptophysin was expressed in case 1 (IHC; magnification, ×200).

The urinary bladder and prostate cases showed fundamentally the same histopathological features. Diffuse proliferation of the small-sized neoplastic cells with occasional geographic necrosis was observed. These tumor cells had scant cytoplasm and round to oval hyperchromatic nuclei without nucleoli (Fig. 2A and B). Mitotic figures and apoptotic bodies were frequently observed (Fig. 2A and B). Direct invasion into the urinary bladder or the prostate portion of the urethra was observed in cases 3 and 4.

A carcinoma *in situ* component was present in the surrounding bladder mucosa in case 1 (Fig. 2A, inset); however, no other components were detected in case 2. A conventional adenocarcinoma component was present in the two prostate cases (Fig. 2B).

### Immunohistochemical findings

Synaptophysin and CD56 were diffusely expressed in all four cases (Fig. 2C); however, none of these cases presented positive immunoreactivity for chromogranin A.

## Discussion

The cytological examination of urine specimens is important in the detection, diagnosis and follow-up of patients with bladder cancer. It is well-recognized that cytological examination of urine specimens has high sensitivity for detection of conventional high-grade urothelial carcinoma (10). Additionally, the cytological features of rare histopathological variants of urothelial carcinoma, such as micropapillary, nested and sarcomatoid, have also been described (11–16). However, prostate cancer cells are rarely identified in the urinary cytological specimen (17), and it has been recognized that routine examination of urine cytology for detection of prostate cancer is not recommended due to low sensitivity (18).

The cytological features of previously reported cases of small cell carcinoma of the urinary bladder are as follows: i) isolated single cells or small groups of the neoplastic cells are present in a hemorrhagic and inflammatory background; ii) nuclear molding is characteristic; iii) the tumor cells are small to medium in size with scant cytoplasm and have a high nuclear/cytoplasmic ratio; and iv) the nuclei are hyperchromatic containing finely granular chromatin without inconspicuous nucleoli (3–8). These cytomorphological features correspond to those of pulmonary small cell carcinoma and are also the same as those of the previously reported cytological features of small cell carcinoma of the prostate (9). The cytological features of the current four cases of small cell carcinoma of the urinary bladder and prostate are identical to the abovementioned features.

Small cell carcinoma of the bladder frequently has another histopathological component, which is present in 40–50% of cases (19). The most common component is a conventional urothelial carcinoma, including carcinoma *in situ*, followed by squamous cell carcinoma and adenocarcinoma (1). Case 1 in the present study had a carcinoma *in situ* component. Therefore, other carcinoma components, such as conventional high-grade urothelial component, can be detected in the urinary cytological specimen; although, a conventional urothelial carcinoma component was not observed in the urinary cytological specimen of case 1. von Hoeven and Artymyshyn reported the cytological features of 13 cases of small cell carcinoma of the urinary bladder (6). The cases included nine cases of mixed small cell carcinoma: One case of urothelial dysplasia, three cases of carcinoma *in situ*, three cases of invasive urothelial carcinoma, one case of adenocarcinoma, and one case of adenocarcinoma and squamous cell carcinoma. Among 31 urine specimens from those mixed small cell carcinoma cases, only nine specimens had exclusively non-small cell type carcinoma cells, and the remaining 22 specimens included only small cell carcinoma components (6). In contrast, Acs *et al* reported the cytological characteristics of six cases of small cell carcinoma of the urinary bladder (3). In five of the cases, the majority of tumor cells in the urine specimens belonged to conventional urothelial cell carcinoma component, leading to a cytodiagnosis of urothelial carcinoma in these cases (3). Therefore, the authors claimed that the presence of conventional urothelial carcinoma component can mask the coexisting small cell carcinoma component (3). Moreover, high-grade urothelial carcinoma may occasionally resemble small cell carcinoma. However, the presence of cellular pleomorphism and prominent nucleoli, as well as lack of nuclear molding, aid in distinguishing conventional urothelial carcinoma from small cell carcinoma (3,5). Small cell carcinoma shows characteristic cytological features; thus, careful observation of the urinary cytological specimen can lead to a correct diagnosis even if a conventional urothelial carcinoma component is present, since this type of tumor shows a highly aggressive clinical course and early diagnosis is important (1).

In addition, small cell carcinoma of the prostate frequently has a conventional adenocarcinoma component. According to the largest study of this type of tumor reported by Wang and Epstein, pure small cell carcinoma was observed in 54 of 95 (57%) cases with the remaining cases admixed with conventional prostate adenocarcinoma (2). The current two prostate cases also had a conventional adenocarcinoma component. However, conventional adenocarcinoma was not detected in the urine specimen in the two cases. It is speculated that conventional adenocarcinoma components were present within the prostate, whereas small cell carcinoma components had directly invaded into the urinary bladder or the prostatic portion of the urethra in the two cases, resulting in the presence of only the small cell carcinoma component in the urine specimen. Small cell carcinoma of the prostate also shows an aggressive clinical course (20), thus early detection is required for appropriate treatment. This report indicates that a cytodiagnosis of this type of tumor may be possible since it frequently invades into the urinary bladder or prostatic portion of the urethra.

In conclusion, we described the cytological features of four cases of small cell carcinoma of the urinary bladder and prostate. The cytological features of this type of tumor are characteristic. Therefore, careful observation of the urine specimen can lead to a correct diagnosis of small cell carcinoma of the urinary bladder. In addition, a cytodiagnosis of prostate small cell carcinoma may also be possible.

## Figures and Tables

**Table I:** Clinicopathological features of small cell carcinoma of the urinary bladder and prostate.

				Cytological features			Histopathological and immunohistochemical features	
					
Case no.	Age (years)	Gender	Location	Background	Number of tumor cells	Cellular arrangement	Other component	Immunohistochemical features
1	77	Male	Urinary bladder	Clean	Many	Small clusters	Carcinoma *in situ*	Syn(+), CD56(+), Chr(−)
2	82	Male	Urinary bladder	Inflammatory	A few	Small clusters	None	Syn(+), CD56(+), Chr(−)
3	64	Male	Prostate	Inflammatory	Many	Small clusters	Adenocarcinoma	Syn(+), CD56(+), Chr(−)
4	73	Male	Prostate	Inflammatory	A few	Small clusters to single	Adenocarcinoma	Syn(+), CD56(+), Chr(−)

Chr, chromogranin A; Syn, synaptophysin.
